# Estimated glomerular filtration rate predicts incident stroke among Ghanaians with diabetes and hypertension

**DOI:** 10.1016/j.jns.2018.11.017

**Published:** 2019-01-15

**Authors:** Fred Stephen Sarfo, Linda Meta Mobula, Osei Sarfo-Kantanka, Sheila Adamu, Jacob Plange-Rhule, Daniel Ansong, Rexford Adu Gyamfi, James Duah, Bertha Abraham, David Ofori-Adjei

**Affiliations:** a Department of Medicine, Kwame Nkrumah University of Science & Technology, Kumasi, Ghana; b Department of Medicine, Komfo Anokye Teaching Hospital, Kumasi, Ghana; c Johns Hopkins University School of Medicine, Baltimore, MD, USA; d Johns Hopkins University Bloomberg School of Public Health, Baltimore, MD, USA; e Ghana College of Physicians and Surgeons, Accra, Ghana; f Agogo Presbyterian Hospital, Agogo, Ghana; g Kings Medical Center, Botanga, Ghana; h Atua Government Hospital, Somanya, Ghana; i Department of Medicine & Therapeutics, University of Ghana, School of Medicine and Dentistry, Accra, Ghana

**Keywords:** eGFR, Stroke risk, West Africa, Chronic kidney disease, APOL-1

## Abstract

**Background:**

Sub-Saharan Africa is currently experiencing a high burden of both chronic kidney disease (CKD) and stroke as a result of a rapid rise in shared common vascular risk factors such as hypertension and diabetes mellitus. However, no previous study has prospectively explored independent associations between CKD and incident stroke occurrence among indigenous Africans. This study sought to fill this knowledge gap.

**Methods:**

A prospective cohort study involving Ghanaians adults with hypertension or type II diabetes mellitus from 5 public hospitals. Patients were followed every 2 months in clinic for 18 months and assessed clinically for first ever stroke by physicians. Serum creatinine derived estimated glomerular filtration rates (eGFR) were determined at baseline for 2631 (81.7%) out of 3296 participants. We assessed associations between eGFR and incident stroke using a multivariate Cox Proportional Hazards regression model.

**Results:**

Stroke incidence rates (95% CI) increased with decreasing eGFR categories of 89, 60–88, 30–59 and <29 ml/min corresponding to incidence rates of 7.58 (3.58–13.51), 14.45 (9.07–21.92), 29.43 (15.95–50.04) and 66.23 (16.85–180.20)/1000 person-years respectively. Adjusted hazard ratios (95%CI) for stroke occurrence according to eGFR were 1.42 (0.63–3.21) for eGFR of 60-89 ml/min, 1.88 (1.17–3.02) for 30-59 ml/min and 1.52 (0.93–2.43) for <30 ml/min compared with eGFR of >89 ml/min. Adjusted HR for stroke occurrence among patients with hypertension with eGFR<60 ml/min was 3.69 (1.49–9.13), p = .0047 and among those with diabetes was 1.50 (0.56–3.98), p = .42.

**Conclusion:**

CKD is dose-dependently associated with occurrence of incident strokes among Ghanaians with hypertension and diabetes mellitus. Further studies are warranted to explore interventions that could attenuate the risk of stroke attributable to renal disease among patients with hypertension in SSA.

## Highlights

• We assessed association between incident stroke and estimated glomerular filtration rate.
• 2631 participants stroke-free Ghanaian adults with hypertension or diabetes were followed for 14 months.
• There were 45 incident strokes.
• Incident stroke risk independently increased with declining eGFR.

## Graphical abstract

**Figure:**
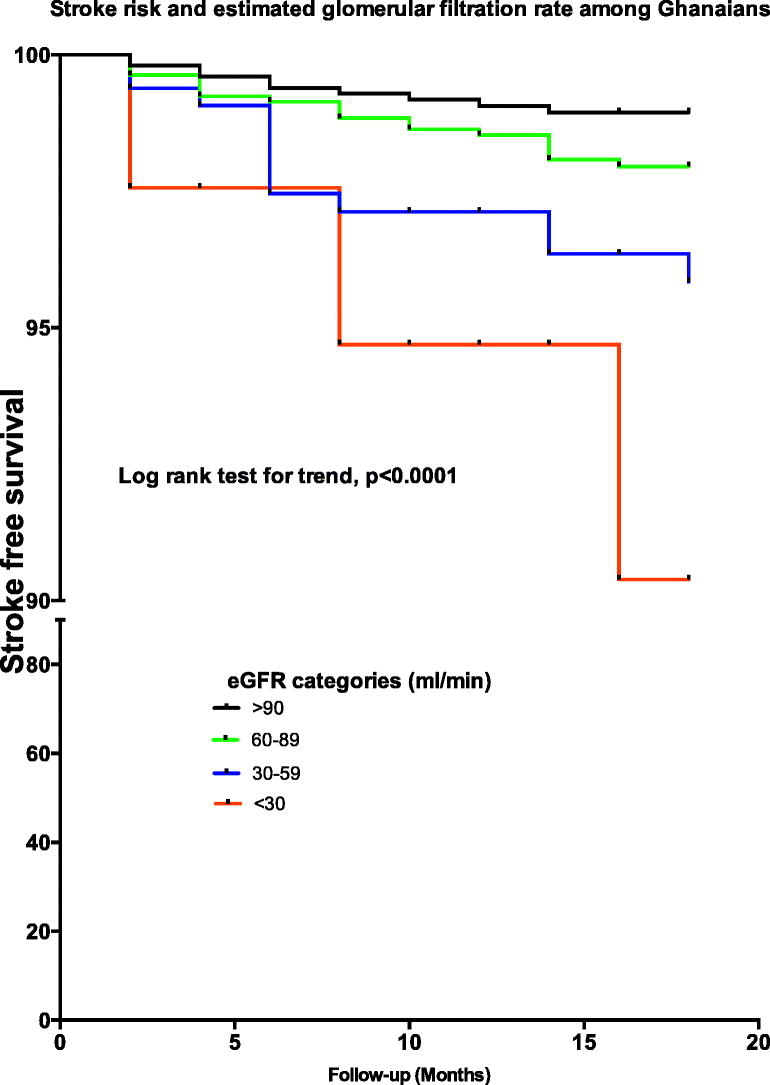

## 1 Introduction

The incidence, prevalence and mortality secondary to stroke in Low-and-Middle Income Countries (LMICs) in sub-Saharan Africa (SSA) have rapidly risen in recent decades [[1], [2], [3], [4], [5], [6], [7], [8], [9], [10], [11], [12], [13], [14]]. These recent secular trends contrasts sharply with the scenario in High-Income Countries (HICs) where burden of stroke is receding due to improved control of vascular risk factors [[15], [16], [17]]. Stroke among Africans has a predilection to be hemorrhagic [18], affect a younger population [19] and is associated with a myriad of post-stroke comorbidities including persisting functional limitations [20], depression [21,22], cognitive impairment [23,24], and stigma [25] in the setting with limited access to neurologists and rehabilitation services. Because stroke care is severely challenged in LMICs, the most sustainable long-term approach to mitigating the enormous burden imposed by stroke in LMICs is to identify and characterize the risk factors associated with stroke occurrence for primary prevention at the population level [[26], [27], [28]].

The association between chronic kidney disease (CKD) and stroke occurrence among Africans although some studies have demonstrated the existence of such associations in other populations [[29], [30], [31], [32], [33], [34], [35]]. Pathophysiologically, both the brain and kidneys are susceptible to vasculature injuries due to reliance on similar microvasculature that allows for continuous high volume perfusion. In addition, stroke and renal disease commonly share traditional vascular risk factors such as hypertension, diabetes, obesity, dyslipidemia and obesity. There are suggestions from the literature of regional and racial differences on the effect of CKD on incident stroke, with a greater burden among Asians [36].

There are no published studies among indigenous Africans that have specifically assessed the association between CKD and stroke. The highest frequencies of renal related variants of apolipoprotein L1 (APOL1)-associated with non-diabetic kidney disease are reported among West Africans [37,38]. Given the recently reported association between APOL-1 variants and small vessel disease strokes among West Africans [39], there is justifiable scientific incentive to investigate the associations between CKD and stroke risk among indigenous Africans further. We therefore sought to narrow the existing knowledge gaps by exploring prospectively the association between chronic kidney disease and stroke incidence among Ghanaians with hypertension and/or diabetes mellitus. Participants were recruited as part of a pragmatic clinical trial aimed at improving access to medicines for the control of hypertension and diabetes by offering medications at differential pricing [40].

## 2 Methods

### 2.1 Study design

The Ghana Access and Affordability Program (GAAP) pilot study is a prospective cohort study involving adults with hypertension (HPT), hypertension with diabetes mellitus (HPT + DM) and diabetes mellitus (DM) at five public hospitals in Ghana. The study sites included the Agogo Presbyterian Hospital, (APH), Atua Government Hospital, (AGH), Komfo Anokye Teaching Hospital, (KATH), Kings Medical center, (KMC) and the Tamale Teaching Hospital, (TTH). Ethical approval was obtained from all study sites. The study protocol has been published elsewhere but a brief synopsis is provided below [40].

### 2.2 Evaluation of study participants

Informed consent was obtained from all study participants prior to enrollment into the study. Trained research assistants followed Standard Operation Procedures across sites to collect demographic information including age, gender, educational attainment, employment status; and lifestyle behaviors such as alcohol use, cigarette smoking, level of physical activities, frequency and daily quantities of fruits and vegetable consumption as well as table added salt were assessed through interviews and responses collected on a questionnaire. A detailed medical history including duration of hypertension or diabetes diagnosis and current medications lists taken were obtained. Anthropometric evaluations including measurement of weight, height and waist circumference were performed by Study nurses. Body mass index (BMI) of each participant was then derived by dividing the weight in kilograms by the square of the height in meters [40].

### 2.3 Laboratory measurements

An International Organization for Standardization (ISO)-certified and quality-assured laboratory was contracted to run all biochemical panels which included serum creatinine, lipid profile and hemoglobin A1C for subjects with diabetes. Samples were transported to the laboratory by trained phlebotomists on the same day of collection often within 4 h or where not feasible (KMC and AGH sites), samples were stored in a freezer before transported to the laboratory the next day.

### 2.4 Stroke diagnosis

Stroke diagnosis was based on the World Health Organization definition [41], if participant had ever experienced sudden onset of weakness or sensory loss on one side of the body, sudden loss of vision, or sudden loss of speech. These questions were derived from the 8-item questionnaire for verifying stroke free status (QVSFS) which has been validated locally [[42], [43], [44]]. QVSFS was used as neuro-imaging facilities were not available at any of the study sites at the time of the study. Study participants visited every 2 months for 18 months to assess control of hypertension and diabetes mellitus and to assess for vascular complications including stroke. Stroke diagnosis was adjudicated by FSS, (a neurologist). Prevalent stroke was defined as stroke diagnosis at time of enrollment into the study while incident stroke was defined as stroke diagnosed during follow-up of study participants. Incident stroke was identified prospectively among participants without stroke at baseline.

### 2.5 Renal impairment

Renal impairment was defined using estimated glomerular filtration rate (eGFR) calculated from baseline serum creatinine measurement using the Chronic Kidney Disease Epidemiology Collaboration (CKD-EPI) formula [45].

### 2.6 Physical activity

Individuals were classified as physically active if they were regularly involved in moderate exercise (walking, cycling, or gardening) or strenuous exercise (jogging, football, and vigorous swimming) for 4 h or more per week.

### 2.7 Alcohol use

Alcohol use was categorized into current users (users of any form of alcoholic drinks) or never/former drinker while alcohol intake was categorized as low drinkers (1–2 drinks per day for female and 1–3 drinks per day for male) and high drinker (>2 drinks per day for female and >3 drinks per day for male. 1 drink or 1 unit of alcohol = 8 g of alcohol) [46].

### 2.8 Smoking status

Smoking status was defined as current smoker (individuals who smoked any tobacco in the past 12 months) or never or former smoker [46]. Vegetable and fruit intake was assessed based on number of daily servings per week.

### 2.9 Statistical analysis

Baseline characteristics of patients with hypertension, diabetes and dual diagnosis of diabetes and hypertension were compared using Analysis of variance for 3-group comparisons or by using the Student's *t*-test for 2-group comparisons. Proportions were compared using the Chi-squared tests or Fisher's exact test for proportions with subgroupings <5. At baseline, factors associated with prevalent stroke were assessed using a logistic regression model. Crude incidence rates of stroke according to eGFR were calculated and expressed as events/1000-person years of follow-up and 95%CI calculated using the Mid-P exact test. A multivariate Cox Hazards Proportion regression analysis was fitted to identify factors independently associated with the risk of stroke with the inclusion of eGFR as a categorical variable (>89 ml/min [referent], 60–88 ml/min, 30-59 ml/min, <30 ml/min) in the model. Other independent variables evaluated included age, gender, location of residence, employment status, previous cigarette smoking, current alcohol use, physical activity, table added salt, fruit and vegetable intake, level of healthcare institution (primary, secondary or tertiary), and central obesity. Variable selection was based on clinical and empirical significance of covariates in the model. Patients were censored either the date of stroke, at the last visit for those who died, were lost-to-follow up, and at July 31, 2017 for the remainder. In all analyses, two-tailed p-values < .05 were considered statistically significant. Secondary analyses included assessing association between eGFR of <60 ml/min and stroke risk among participants with hypertension only, hypertension and diabetes mellitus and or diabetes only. Model diagnostics and fit were assessed using residual plots analysis and visual inspection for collinearity of variables in the Cox models. Statistical analysis was performed using SPSS and GraphPad Prism version 7.

## 3 Results

### 3.1 Baseline demographic and clinical characteristics

We enrolled 3296 study participants comprising of 1867 (56.6%) with hypertension, 1006 with both diabetes and hypertension (30.5%) and 422 (12.9%) with diabetes mellitus. The demographics, lifestyle and clinical characteristics are shown in Table 1. Among the entire cohort, mean estimated glomerular filtration rate was 76.6 ± 16.2 ml/min, being lowest among diabetics with hypertension of 74.4 ± 18.4 ml/min followed by 76.6 ± 15.4 ml/min among those with hypertension and 82.5 ± 12.2 ml/min among diabetics, p < .0001. Correspondingly, 19.2% of diabetic hypertensive participants, 14.8% of participants with hypertension and 6.5% of diabetics had eGFR <60 ml/min, p < .0001 (Table 1).

**Table 1:** Baseline demographic and clinical characteristics of Study population.

Characteristic	Hypertension (HPT) only	Diabetes Mellitus (DM) only	Hypertension and Diabetes Mellitus	Overall (*n* = 3296)	P-value	P-value	P-value	P-value
	*N* = 1867	*N* = 422	*N* = 1006		ANOVA	HPT vs DM	HPT vs HPT + DM	DM vs HPT + DM
Age, mean ± SD	58.0 ± 13.0	49.7 ± 12.2	60.0 ± 10.8	57.5 ± 12.7	<0.0001	<0.0001	<0.0001	<0.0001
Female, n (%)	1434 (76.9)	308 (73.0)	778 (77.4)	2520 (76.5)	0.17	0.09	0.77	0.07
Location of residence					<0.0001	<0.0001	<0.0001	0.51
Urban	635 (34.1)	238 (56.4)	564 (56.1)	1437 (43.7)				
Semi-urban	384 (20.6)	112 (26.5)	247 (24.6)	743 (22.6)				
Rural	843 (45.3)	72 (17.1)	195 (19.4)	1110 (33.7)				
Highest Educational status					0.0005	<0.0001	0.19	0.01
No formal education	743 (39.8)	119 (28.3)	361 (36.0)	1223 (37.2)				
Primary level	294 (15.7)	72 (17.1)	172 (17.1)	538 (16.3)				
Secondary level	627 (33.6)	182 (43.2)	347 (34.6)	1156 (35.1)				
Tertiary level or more	203 (10.9)	48 (11.4)	124 (12.4)	375 (11.4)				
Level of Health Institution					<0.0001	<0.0001	<0.0001	0.04
Tertiary referral level	765 (41.0)	353 (83.6)	781 (77.6)	1899 (57.6)				
Secondary/district level	936 (50.1)	66 (15.6)	217 (21.6)	1219 (37.0)				
Primary level	166 (8.9)	3 (0.7)	8 (0.8)	177 (5.4)				
Vascular Risk Factors								
Duration of hypertension, (years)	7.2 ± 7.2	NA	9.1 ± 7.2	7.8 ± 7.3	NA	NA	<0.0001	NA
Duration of diabetes mellitus, mean ± SD (yrs)	NA	8.1 ± 6.0	9.8 ± 7.2	9.3 ± 7.0	NA	NA	NA	<0.0001
Average Systolic Blood Pressure at enrollment (mmHg), mean ± SD	142.5 ± 21.3	125.8 ± 18.3	145.3 ± 22.7	141.2 ± 22.2	<0.0001	<0.0001	0.001	<0.0001
Average Diastolic Blood Pressure at enrollment (mmHg), mean ± SD	83.2 ± 13.1	75.9 ± 10.8	81.8 ± 12.5	81.9 ± 12.9	<0.0001	<0.0001	0.005	<0.0001
Medical co-morbidities								
Previous stroke diagnosis	84 (4.5)	5 (1.9)	70 (7.0)	159 (4.8)	<0.0001	0.002	0.005	<0.0001
Previous heart failure diagnosis	121 (6.5)	10 (2.4)	51 (5.1)	182 (5.5)	0.003	0.001	0.13	0.02
Lifestyle/Behavioral factors								
Current alcohol use	160 (8.6)	26 (6.2)	60 (6.0)	246 (7.5)	0.02	0.10	0.01	0.89
Current cigarette smoking	9 (0.48)	2 (0.47)	5 (0.50)	16 (0.49)	0.99	0.98	0.96	0.95
Previous cigarette smoking	111 (5.9)	29 (6.9)	86 (8.5)	226 (6.9)	0.03	0.47	0.008	0.29
Fruit consumption								
Daily intake of fruits in a week, mean ± SD	2.54 ± 2.01	2.89 ± 2.05	2.61 ± 2.01	2.60 ± 2.04	0.006	0.001	0.41	0.02
Fruit servings per day, mean ± SD	1.64 ± 1.68	1.76 ± 1.34	1.69 ± 1.33	1.67 ± 1.54	0.30	0.16	0.42	0.34
Fruit servings per day, by thirds					<0.0001	<0.0001	<0.0001	0.49
0–1	1145 (61.3)	199 (47.2)	490 (48.7)	1834 (55.7)				
2–4	646 (34.6)	215 (50.9)	488 (48.5)	1349 (40.9)				
>5	76 (4.1)	8 (1.9)	28 (2.8)	112 (3.4)				
Vegetable consumption								
Daily intake of vegetables in a week, mean ± SD	5.00 ± 2.20	4.72 ± 2.14	4.86 ± 2.11	4.92 ± 2.17	0.04	0.02	0.10	0.28
Vegetable servings per day, mean ± SD	2.19 ± 1.60	2.28 ± 1.41	2.28 ± 1.50	2.23 ± 1.55	0.27	0.29	0.15	0.98
Vegetable servings per day, by thirds					0.007	0.03	0.01	0.27
0–1	618 (34.5)	115 (28.0)	287 (29.3)	1020 (32.0)				
2–4	1061 (59.2)	263 (64.0)	638 (65.0)	1962 (61.6)				
>5	112 (6.3)	33 (8.0)	56 (5.7)	201 (6.3)				
Added salt at table					<0.0001	0.03	<0.0001	0.0006
Never	1491 (79.9)	326 (77.4)	866 (86.1)	2683 (81.5)				
Rarely	157 (8.4)	26 (6.2)	42 (4.2)	225 (6.8)				
occasionally	117 (6.3)	33 (7.8)	54 (5.3)	204 (6.2)				
Very often	101 (5.4)	36 (8.6)	44 (4.4)	181 (5.5)				
Regular Physical activity								
Proportions of subjects engaging in physical activities daily	1092 (58.5)	294 (69.7)	640 (61.5)	2026 (61.5)	<0.0001	<0.0001	0.008	0.03
Duration of time spent on physical activities in minutes, mean ± SD	18.2 ± 23.0	24.3 ± 26.7	20.5 ± 23.9	19.7 ± 23.9	<0.0001	<0.0001	0.01	0.008
					0.002	0.0005	0.07	0.08
>60 min	141 (7.6)	53 (12.6)	88 (8.7)	282 (8.6)				
20–59 min	723 (38.7)	176 (41.7)	422 (41.9)	1321 (40.1)				
<20 min	1003 (53.7)	193 (45.7)	496 (49.3)	1692 (51.4)				
Anthropometric Indicators								
Waist circumference, mean ± SD	95.0 ± 13.2	92.0 ± 12.0	98.2 ± 12.6	95.6 ± 13.0	<0.0001	<0.0001	<0.0001	<0.0001
Waist circumference elevated, n (%)	1075 (57.9)	226 (53.8)	704 (70.1)	2005 (61.1)	<0.0001	0.13	<0.0001	<0.0001
Body Mass Index, mean ± SD	26.9 ± 5.9	24.8 ± 4.6	26.7 ± 5.2	26.6 ± 5.6	<0.0001	<0.0001	0.42	<0.0001
					<0.0001	<0.0001	0.02	<0.0001
BMI <18.5 kg/m^2^	66 (3.6)	32 (7.7)	32 (3.2)	130 (4.0)				
BMI 18.5–24.9 kg/m^2^	737 (40.1)	196 (47.5)	375 (37.9)	1308 (40.3)				
BMI 25.0–29.9 kg/m^2^	549 (29.8)	125 (30.3)	351 (35.5)	1025 (31.6)				
BMI > 30 kg/m^2^	488 (26.5)	60 (14.5)	231 (23.4)	779 (24.0)				
Laboratory Indicators								
Serum creatinine, mean ± SD	82.1 ± 55.7	71.4 ± 26.1	83.8 ± 54.7	81.3 ± 52.8	0.0008	0.0005	0.45	<0.0001
eGFR, mean ± SD	76.6 ± 15.4	82.5 ± 12.2	74.4 ± 18.4	76.6 ± 16.2	<0.0001	<0.0001	0.003	<0.0001
Proportions with eGFR cut-off's								
eGFR 60–89 ml/min, n (%)	1321 (85.2)	304 (93.5)	658 (80.6)	2283 (84.8)	<0.0001	0.0006	<0.0001	<0.0001
eGFR 30–59 ml/min, n (%)	211 (13.6)	19 (5.9)	128 (15.7)	358 (13.3)				
eGFR 15–29 ml/min, n (%)	9 (0.6)	2 (0.6)	24 (2.9)	35 (1.3)				
eGFR <15 ml/min, n (%)	9 (0.6)	0 (0.0)	6 (0.7)	15 (0.6)				
HBA1C (%), mean ± SD	NA	9.3 ± 2.7	8.5 ± 2.4	8.6 ± 2.6	NA	NA	NA	<0.0001
Proportion with HBA1C <7%, n (%)	NA	70 (21.0)	275 (33.7)	345 (30.0)	NA	NA	NA	<0.0001
Antihypertensive Medications, mean ± SD	2.2 ± 0.9	N/A	1.8 ± 0.9	2.0 ± 0.9	N/A	N/A	<0.0001	N/A
Anti-glycemic Medications, mean ± SD	N/A	2.2 ± 0.8	2.2 ± 0.8	2.2 ± 0.8	N/A	N/A	N/A	0.99
Statins, n (%)	89 (4.8)	49 (11.6)	191 (19.0)	329 (10.0)	<0.0001	<0.0001	<0.0001	0.0007
Antiplatelets, n (%)	134 (7.2)	36 (8.5)	164 (16.3)	334 (10.1)	<0.0001	0.34	<0.0001	0.0001

### 3.2 Association between eGFR and Incident stroke

**Fig. 1:**
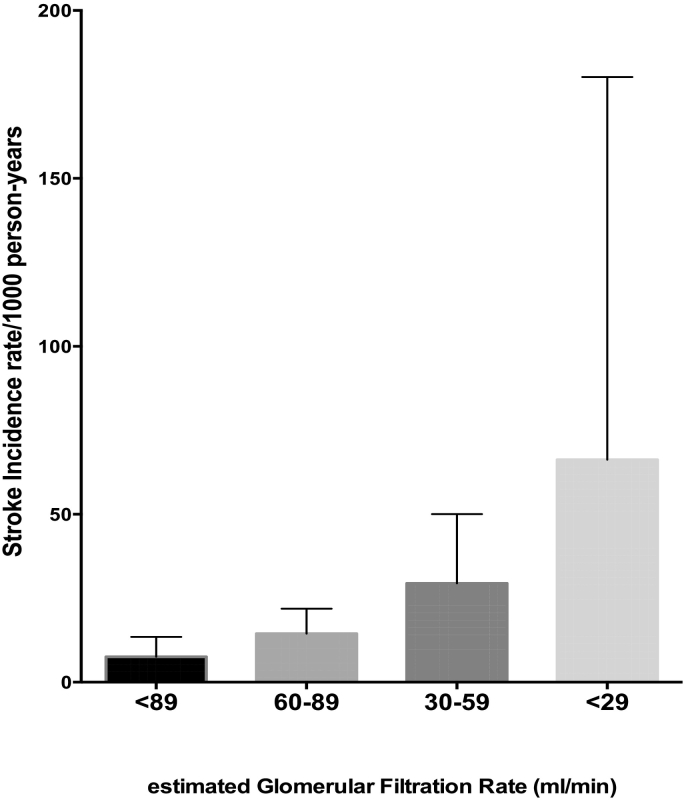
Incidence rates of stroke according to baseline estimated glomerular filtration rates among Ghanaians with hypertension and or diabetes. Each block represents incidence rate/1000 person years and bars represent upper limits of 95% Confidence intervals.

There were 45 incident strokes during follow-up among 2631 participants who did not have baseline history of stroke and had baseline eGFR measurements. Participants with incident strokes are compared with those without stroke in Table 2. The mean duration of follow-up per participant was 14.6 ± 5.6 months with 1548 (58.9%) completing month 18 visit contributing to 3200 person-years of follow-up. Stroke incidence rates (95% CI) increased with decreasing eGFR categories of 89, 60–88, 30–59 and <29 ml/min corresponding to stroke incidence rates of 7.58 (3.58–13.51), 14.45 (9.07–21.92), 29.43 (15.95–50.04) and 66.23 (16.85–180.20)/1000 person-years respectively (Fig. 1). In a model specifying eGFR as categories with >89 ml/min as referent, eGFR level of 60-89 ml/min had adjusted hazards ratio of 1.42 (0.63–3.21), 30–59 ml/min had aHR of 1.88 (1.17–3.02) and eGFR < 29 ml/min had aHR of 1.52 (0.93–2.43). The only other factor that remained significantly associated with incident stroke was previous history of cigarette smoking with aHR of 2.70 (1.11–6.57) (Table 3). Use of an ACE-inhibitor was associated with unadjusted HR of 1.05 (0.58–1.89), p = .88, and use of ARB was associated with unadjusted HR of 1.36 (0.73–2.54), p = .33 for incident strokes and were therefore not included in final adjusted models.

**Table 2:** Demographic and clinical characteristics of participants with incident stroke versus those without stroke.

	Stroke, *N* = 45	No stroke, *n* = 2586	P-value
Age, mean ± SD	61.7 ± 10.8	57.1 ± 12.4	0.02
Male gender, n (%)	17 (37.8)	562 (21.7)	0.01
Disease class, n (%)			0.11
Hypertension only	20 (44.4)	1489 (57.6)	
Type 2 Diabetes Mellitus only	5 (11.1)	317 (12.3)	
Both Hypertension and T2DM	20 (44.5)	780 (30.1)	
Level of institution, n (%)			0.01
Tertiary	32 (71.1)	1348 (52.1)	
Secondary	10 (22.2)	1142 (44.2)	
Primary	3 (6.7)	96 (3.7)	
Location of residence, n (%)			0.20
Urban	21 (46.7)	1029 (39.8)	
Peri-urban	13 (28.9)	590 (22.8)	
Rural	11 (24.4)	967 (37.4)	
Educational attainment, n (%)			0.67
Tertiary	5 (11.1)	286 (11.1)	
Secondary	14 (31.1)	893 (34.5)	
Primary	11 (24.4)	451 (17.4)	
None	15 (33.4)	956 (37.0)	
Unemployed, n (%)	21 (46.7)	761 (29.4)	0.01
National Health Insurance cover for all medicines, n (%)	24 (53.3)	1346 (52.0)	0.86
Monthly Income levels, n (%)			0.82
>1000 GHS	2 (4.4)	207 (8.0)	
210–1000 GHS	13 (28.9)	719 (27.8)	
<210 GHS	16 (35.6)	942 (36.4)	
Unknown	14 (31.1)	718 (27.8)	
Cigarette use			0.0006
Current use	0 (0.0)	11 (0.4)	
Former use	9 (20.0)	156 (6.0)	
Never use	36 (80.0)	2419 (93.6)	
Current alcohol use, n (%)	4 (8.9)	216 (8.4)	0.90
Table added salt, n (%)	6 (13.3)	463 (17.9)	0.43
Physical inactivity, n (%)	22 (48.9)	940 (36.3)	0.08
Fruit intake: daily servings/week, mean ± SD	2.2 ± 1.7	2.7 ± 2.1	0.18
Fruit intake daily servings/week, n (%)			0.06
0	6 (13.3)	369 (14.3)	
1 to 3	33 (73.3)	1475 (57.0)	
4 to 7	6 (13.4)	742 (28.7)	
Vegetable intake: daily servings/week, mean ±	4.9 ± 2.0	5.1 ± 2.1	0.46
Vegetable intake, n (%)			0.34
1 to 2	9 (20.0)	385 (14.9)	
>3	36 (80.0)	2201 (85.1)	
Heart failure, n (%)	4 (8.9)	158 (6.1)	0.44
Body mass index, mean ± SD kg/m^2^	25.8 ± 5.1	26.6 ± 5.5	0.34
Raised waist circumference, n (%)	20 (44.4)	1611 (62.3)	0.01
Duration of Hypertension (years)			
Mean ± SD	8.3 ± 6.6	7.6 ± 7.1	0.54
Median (IQR)	7.0 (3.0–12.0)	6.0 (3.0–10.0)	0.26
Duration of Diabetes (years)			
Mean ± SD	9.8 ± 5.8	9.1 ± 6.8	0.61
Median (IQR)	11.0 (4.5–15.0)	8.0 (4.0–12.5)	0.32
Antihypertensive medications, mean ± SD	1.9 ± 1.0	1.9 ± 1.0	0.61
Anti-hypertensive medications			
ACE-I	19 (42.2)	1048 (40.5)	0.82
ARB	15 (33.3)	663 (25.6)	0.24
Beta blocker	4 (8.9)	229 (8.9)	0.99
Calcium channel blocker	34 (75.6)	1741 (67.3)	0.24
Diuretics	8 (17.8)	751 (29.0)	0.10
Methyl dopa	6 (13.3)	344 (13.3)	0.99
Hydrallazine	1 (2.2)	23 (0.9)	0.35
Statin	7 (15.6)	247 (9.6)	0.18
Aspirin	8 (17.8)	270 (10.4)	0.11
eGFR, (ml/min) mean ± SD	67.4 ± 21.9	76.9 ± 16.0	<0.0001

ACE-I = Angiotensin-converting enzyme inhibitor; ARB = Angiotensin Receptor Blocker, eGFR = estimated glomerular filtration rate derived using the CKD-EPI formula from serum creatinine measurement (data available for 2631 participants).

**Table 3:** Predictors of Incident Stroke Among a Prospective cohort of Ghanaians with Hypertension and/or Diabetes Mellitus.

Predictors	Unadjusted HR	P-value	Adjusted HR	P-value
Male gender	2.27 (1.25–4.16)	0.007	1.26 (0.55–2.89)	0.59
Age/10 year increase	1.36 (1.05–1.75)	0.02	1.04 (0.78–1.39)	0.79
Tertiary level	0.83 (0.46–1.50)	0.53	–	
Secondary level	0.23 (0.06–0.84)	0.03	–	
Primary level	1		–	
Location				
Urban	1.38 (0.96–1.98)	0.09	–	
Semi-urban	2.00 (0.89–4.45)	0.09	–	
Rural	1			
Hypertension	0.96 (0.59–1.56)	0.86	–	
Diabetes and hypertension	1.54 (0.58–4.10)	0.39	–	
Diabetes	1		–	
Unemployed	2.06 (1.15–3.70)	0.02	1.60 (0.82–3.13)	0.17
Previous Cigarette smoking	3.67 (1.77–7.62)	0.0005	2.70 (1.11–6.57)	0.03
Alcohol	1.04 (0.37–2.91)	0.94	–	
Physical inactivity	1.78 (0.99–3.19)	0.05	1.65 (0.91–3.01)	0.10
Vegetable intake	0.94 (0.82–1.07)	0.37	–	
Fruit intake	0.90 (0.77–1.06)	0.20	–	
eGFR categories (ml/min)				
>89	1		1	
60–88	1.84 (0.86–3.96)	0.12	1.42 (0.63–3.21)	0.4
30–59	1.97 (1.30–2.97)	0.001	1.88 (1.17–3.02)	0.009
<30	2.03 (1.32–3.12)	0.001	1.52 (0.93–2.48)	0.10
Increased Waist circumference	0.49 (0.27–0.89)	0.02	0.66 (0.33–1.33)	0.25

### 3.3 Secondary analyses

Unadjusted HR for stroke occurrence among those with hypertension only was 4.87 (2.02–11.76), p < .0004 for an eGFR <60 ml/min and that for those with diabetes was 1.83 (95% CI of 0.73–4.59), p = .20. The association between eGFR and stroke risk remained significant among participants with hypertension only, adjusted HR of 3.69 (1.49–9.13), p = .0047 for those with eGFR<60 ml/min. However among those with diabetes and hypertension, the adjusted HR was 1.32 (0.46–3.78), p = .61 and among those with diabetes only, adjusted HR was 4.00 (0.33–48.36), p = .28 (Table 4). Those with any diabetes (diabetes only or diabetes with hypertension), adjusted HR for stroke occurrence was 1.50 (0.56–3.98), p = .42 for an eGFR<60 ml/min.

**Table 4:** Adjusted Hazards Ratios of factors associated with stroke occurrence according to Hypertension, Diabetes with Hypertension or Diabetes only.

Variables	Hypertension only, *n* = 1506		Diabetes and Hypertension, *n* = 797		Diabetes only, *n* = 323	
	Adjusted HR (95% CI)	p-Value	Adjusted HR (95% CI)	p-Value	Adjusted HR (95% CI)	p-Value
Age, each 10 year increase	1.03 (0.67–1.57)	0.90	1.18 (0.72–1.95)	0.60	0.80 (0.35–1.83)	0.60
Male gender	1.88 (0.67–5.27)	0.23	0.95 (0.28–3.22)	0.57	1.89 (0.21–17.19)	0.57
Unemployed	2.34 (0.85–6.43)	0.10	1.01 (0.37–2.78)	0.40	2.19 (0.35–13.69)	0.40
Previous cigarette smoking	3.67 (1.01–13.42)	0.05	2.56 (0.64–10.30)	0.44	2.87 (0.19–42.43)	0.44
Physical inactivity	3.63 (1.38–9.53)	0.009	0.63 (0.23–1.76)	0.13	4.00 (0.64–24.86)	0.14
eGFR < 60 ml/min	3.69 (1.49–9.13)	0.005	1.32 (0.46–3.78)	0.61	4.00 (0.33–48.36)	0.28

## 4 Discussion

In this prospective hospital based cohort of Ghanaians with hypertension and diabetes mellitus, we found CKD assessed using estimated glomerular filtration rate to be independently associated with stroke occurrence. At baseline, we observed a high frequency of renal impairment among the cohort with approximately 15% having eGFR <60 ml/min. Incident stroke risk increased with declining renal function among a clinically stroke-free cohort at enrollment who were followed up prospectively. Overall, stroke risk increased by 42% among participants with eGFR of 60 to 89 ml/min, by 88% among those with eGFR between 30 and 59 ml/min and by 52% among those with eGFR<30 ml/min compared with participants with normal eGFR. Among patients with hypertension, an eGFR<60 ml/min was significantly associated with an adjusted HR of 3.69 (95%CI: 1.49–9.31) for stroke occurrence while that among diabetic patients did not reach statistical significance. This to the best of our knowledge is the first study in sub-Saharan Africa to prospectively assess the predictive associations between renal dysfunction and stroke risk among individuals on treatment for hypertension and/or diabetes.

A meta-analysis performed nearly a decade ago involving a prospective cohort of 285,000 participants from 33 studies (followed up for 3.2 to 15 years) with 7900 strokes demonstrated a 43% increased risk for stroke occurrence for eGFR <60 ml/min compared with those with normal baseline eGFR [33]. In that seminal meta-analysis, general or hypertensive patients with eGFR < 60 ml/min had a 58% increased risk of stroke compared with those with normal baseline eGFR and Asians had higher risk of stroke from CKD than non-Asians but no African cohorts were included in that study [33]. In the present study, Ghanaian hypertensive patients with eGFR < 60 ml/min had a 269% higher risk of stroke, p = .0047 but no significant association were observed among diabetics. The toxic bidirectional relationship between CKD and cerebrovascular diseases, known as cerebro-renal interaction has gathered scientific momentum over the past decade. CKD is independently associated with both ischemic and hemorrhagic stroke types [[29], [30], [31], [32], [33], [34], [35]] as well as with recurrent strokes [34]. CKD is associated with cerebral microbleeds, which are clinically silent, discrete punctate hypodense lesions 5–10 mm in size evidenced on gradient-recall echo (GRE) T2*-weighted MRI [47]. Cerebral microbleeds are important harbingers of intracerebral hemorrhage [48,49] which are commoner among indigenous Africans and African Americans than European Americans [50,51]. Interestingly, declining renal function is closely associated with a surge in vascular inflammation, oxidative stress and anemia which may potentiate the occurrence of cardiovascular events via generalized endothelial dysfunction and systematic vascular remodeling [52,53]. The salience of renal impairment and increased stroke risk particularly among individuals of African ancestry may have strong genetic underpinnings based on more recent evidence [39]. The SIREN investigators involved in the largest study on stroke in Africa to date, were the first to demonstrate an association between APOL1 variant rs73885319 and small vessel disease (lacunar) strokes among West Africans [39]. Among African Americans, the APOL1 gene has been propounded as a risk locus for chronic kidney disease [54] and West Africans, among whom small vessel disease strokes are the commonest stroke phenotype, have the highest frequencies of APOL1-associated kidney variants on the globe with the Akans of Ghana [55] reporting 43.6% and that among the Yorubas of Nigeria [37,38] being 34.2%. However, it is worth emphasizing the conflicting reports of associations between APOL1 renal related variants and occurrence of strokes from large prospective studies. While the Jackson Heart Study (JHS) [56], Women's Health Initiative (WHI) and Cardiovascular Health Study (CHS) [57] found evidence linking APOL1 variants to adverse CVD events such as strokes, the Systolic Blood Pressure Intervention Trial (SPRINT) [58], the Atherosclerosis Risk in Communities (ARIC) [59], and African American Study of Kidney Disease and Hypertension (AASK) [60] in contrast found no such evidence highlighting the need for further studies to resolve this conundrum.

Our findings have important clinical and public health implications in SSA which now bears a rapidly growing burden of hypertension, diabetes, CKD and stroke globally. Strategic investments in primary prevention of stroke are of paramount importance, given the dire limitations in funding for health infrastructure and availability of skilled personnel to managed CVD in Africa. Regular monitoring of renal function of patients with hypertension and/or diabetes mellitus may be a cost effective strategy in SSA given the dose-response association observed between stages of CKD and risk of stroke occurrence in the present study. This will help identify individuals at high risk of stroke for intensification of control of blood pressure or blood glucose, and reduction of proteinuria to avert the progression of kidney disease and occurrence of CVDs. Health systems should be strengthened to support the use of evidence-based interventions such as Renin-Angiotensin-Aldosterone-System modulators and statins [61] in SSA. A limited sub-analysis performed in the present cohort did not find evidence to support moderation of stroke risk from the use of either ACE-inhibitors or Angiotensin Receptor blockers. This result should however be interpreted with caution given that the indications for use of ACEI or ARBs among participants in the present study were unknown. A systematic review of ischemic stroke survivors on either ACE inhibitors or ARBs showed a modest reduction in risk for recurrent strokes [62] as a secondary prevention strategy, thus a larger sample size may be required to show benefit or otherwise of ACE-I and ARBs in primary prevention of stroke in SSA. Importantly, as the weight of evidence supporting the plausibility of CKD as a risk factor for stroke and indeed other CVDs becomes substantial, the question of whether eGFR is simply a risk marker or is etiologically linked to stroke occurrence will require further interrogation. For instance, among Ghanaians in the present cohort, eGFR was independently associated with poor glycemic [63] and blood pressure control [64], and poor BP control is potently associated with stroke occurrence [65]. However, an interesting nationwide cohort in Taiwan found evidence to suggest that CKD is a causal risk factor for stroke beyond traditional cardiovascular risk factors [66].

The prospective design of our study is an important strength because it minimizes confounding from recall bias, selection bias and reverse causality associations. Furthermore, participants of the study were recruited from hospitals at primary, secondary and tertiary cadres of healthcare distributed across Ghana, which enhances the generalizability of our study findings. However, the lack of confirmation of stroke with neuro-imaging, which is considered as the gold standard is a limitation worth noting. We relied on clinical assessments by study physicians to confirm stroke diagnosis which may be subject to diagnostic misclassification. Unavailability of CT scans at most of the study sites precluded us from radiologic confirmation of clinically suspected strokes. There is a possibility that we missed some severe or fatal stroke cases who did not report to clinic for follow-up since strokes were assessed only among participants who reported for follow-up visits. However, when participants missed clinic visits we followed up with telephone calls to ascertain reasons for default. Well known vascular risk factors such as atrial fibrillation and dyslipidemia were not systematically assessed because electrocardiography were not undertaken for all participants and only a fourth of study population was supported by the study to cover lipid panel costs. In spite of these limitations, we believe our study contributes to literature by providing evidence from West Africa to strengthen the reported associations between chronic kidney disease and stroke occurrence on the globe. More cohort studies of such nature are needed to assess the impact of renal disease on strokes occurrence in low-and-middle income countries in SSA.

In conclusion, chronic kidney disease is dose-dependently associated with occurrence of incident strokes among Ghanaians with hypertension and diabetes mellitus. Further studies are required to explore interventions that could attenuate the risk of stroke attributable to renal disease among patients with hypertension in SSA.

## Contributors

FSS, LMM, JPR, DA and DO-A designed the study and planned analyses, and FSS wrote the first draft of the report. FSS performed statistical analyses. All authors contributed to the collection of data, discussions and interpretation of the data, and to writing of the manuscript. FSS had full access to the data. All authors reviewed and approved drafts of the report.

## Declarations

The authors do not have any competing interests.

Funding for this study was provided by MSD, Novartis, Pfizer, Sanofi (each a Participant Company) and the Bill and Melinda Gates Foundation (collectively, the Funders) through the New Venture Fund (NVF).

The NVF is a not-for-profit organization exempt as a public charity under section 501(c) [3] of the United States Internal Revenue Code of 1986, and assumes financial management of the study as a fiduciary agent and primary contractor for the Funders.

Consistent with anti-trust laws that govern industry interactions, each Participant Company independently and voluntarily will continue to develop its own marketing and pricing strategies reflecting, among other factors, the Company's product portfolios and the patients it serves. For the avoidance of doubt, the Participant Companies committed not to: (i) discuss any price or marketing strategy that may involve any Project-related product; or (ii) make any decision with respect to the presence, absence or withdrawal of any Participant Company in or from any therapeutic area; or (iii) discuss the launching, maintaining or withdrawing of any product in any market whatsoever. Each Participant Company is solely responsible for its own compliance with applicable anti-trust laws.

The Funders were kept apprised of progress in developing and implementing the study program in Ghana but had no role in study design, data collection, data analysis or in study report writing.

FSS was supported by National Institute of Health-National Institute of Neurological Disorders & Stroke; R21 NS094033.
